# Mechanisms and consequences of casein kinase II and ankyrin-3 regulation of the epithelial Na^+^ channel

**DOI:** 10.1038/s41598-021-94118-3

**Published:** 2021-07-16

**Authors:** Tarek Mohamed Abd El-Aziz, Antonio G. Soares, Elena Mironova, Nina Boiko, Amanpreet Kaur, Crystal R. Archer, James D. Stockand, Jonathan M. Berman

**Affiliations:** 1 Department of Cellular and Integrative Physiology, University of Texas Health Science Center At San Antonio, San Antonio, TX 78229-3900 USA; 2 Zoology Department, Faculty of Science, Minia University, El-Minia, 61519 Egypt; 3 Department of Biochemistry, University of Washington, Seattle, Washington 98195 USA; 4 Department of Basic Science, New York Institute of Technology College of Osteopathic Medicine at Arkansas State University, Jonesboro, AR 72401 USA

**Keywords:** Physiology, Cardiovascular biology, Kidney, Neurophysiology, Membrane trafficking, Ion channels, Sodium channels, Kinases, Cellular neuroscience

## Abstract

Activity of the Epithelial Na^+^ Channel (ENaC) in the distal nephron fine-tunes renal sodium excretion. Appropriate sodium excretion is a key factor in the regulation of blood pressure. Consequently, abnormalities in ENaC function can cause hypertension. Casein Kinase II (CKII) phosphorylates ENaC. The CKII phosphorylation site in ENaC resides within a canonical “anchor” ankyrin binding motif. CKII-dependent phosphorylation of ENaC is necessary and sufficient to increase channel activity and is thought to influence channel trafficking in a manner that increases activity. We test here the hypothesis that phosphorylation of ENaC by CKII within an anchor motif is necessary for ankyrin-3 (Ank-3) regulation of the channel, which is required for normal channel locale and function, and the proper regulation of renal sodium excretion. This was addressed using a fluorescence imaging strategy combining total internal reflection fluorescence (TIRF) microscopy with fluorescence recovery after photobleaching (FRAP) to quantify ENaC expression in the plasma membrane in living cells; and electrophysiology to quantify ENaC activity in split-open collecting ducts from principal cell-specific Ank-3 knockout mice. Sodium excretion studies also were performed in parallel in this knockout mouse. In addition, we substituted a key serine residue in the consensus CKII site in β-ENaC with alanine to abrogate phosphorylation and disrupt the anchor motif. Findings show that disrupting CKII signaling decreases ENaC activity by decreasing expression in the plasma membrane. In the principal cell-specific Ank-3 KO mouse, ENaC activity and sodium excretion were significantly decreased and increased, respectively. These results are consistent with CKII phosphorylation of ENaC functioning as a “switch” that favors Ank-3 binding to increase channel activity.

## Introduction

There currently is a worldwide epidemic of hypertension^1^. High blood pressure is a significant risk factor for stroke, heart failure, myocardial infarction, and chronic kidney disease^2^. These manifestations of elevated blood pressure are the leading causes of death worldwide^3^. Nonetheless, we have an incomplete understanding of how blood pressure is controlled. The Epithelial Na^+^ Channel (ENaC) is the final arbiter of Na^+^ excretion in the kidneys^6^. As such, discretionary control of ENaC in principal cells of the aldosterone-sensitive distal nephron fine-tunes renal sodium excretion. Appropriate renal sodium excretion is a key factor in the regulation of blood pressure^4,7^. ENaC serves as one of several key final effectors of the renin-AngII-aldosterone system during regulation of blood pressure^8^. Abnormalities in ENaC function have been directly linked to several human diseases of blood pressure including the hypertensive state of Liddle’s syndrome and the hypotensive state of pseudohypoaldosteronism type I^5,6,9^.

ENaC is a heterotrimeric channel comprised of three distinct subunits, α, β and γ, which share a conserved secondary and tertiary structure^10,11^. Each of the α, β and γ-subunits of ENaC contain short cytosolic NH_2_- and COOH-terminal domains available for interacting with intracellular proteins, two membrane-spanning domains that contribute to the formation of a Na^+^-conducting pore, and a large glycosylated extracellular loop^12^. ENaC activity is regulated by changes in channel open probability (P_o_) and/or by varying the number of channels (N) located in the apical plasma membrane^13–15^.

The COOH-terminal domains of β-ENaC and, to a lesser extent, γ-ENaC are phosphorylated by casein kinase II (CKII)^16^. The physiological importance of this, as well as, the molecular and cellular mechanisms underpinning CKII regulation of ENaC are not well understood. CKII is a heterotetrameric enzyme containing two catalytic α subunits and two regulatory β subunits. CKII is a serine/threonine kinase that is involved in a variety of cellular functions, including DNA damage repair, cell cycle progression and survival^17,18^. Previous work has shown that inhibition of CKII decreases amiloride-sensitive short-circuit currents in isolated mouse trachea and colon; and that mutation of the CKII phosphorylation site in β-ENaC attenuates amiloride-sensitive currents in *Xenopus* oocytes overexpressing recombinant ENaC^19^. Amiloride and its analogs like benzamil block ENaC, and Na^+^ absorption through ENaC is the primary driver of current across the trachea and colon.

Voltage-gated Na^+^ (Na_V_) and KCNQ K^+^ channels are evolutionarily unrelated to ENaC and each other. Nonetheless, possibly due to convergent evolution of key regulatory protein domains, phosphorylation of Na_V_ and KCNQ channels by CKII is critical for their proper subcellular localization and activity^20–22^. CKII phosphorylates Na_V_ and KCNQ channels within a conserved cytosolic ankyrin binding “anchor” motif with phosphorylation acting as a molecular “switch” to facilitate ankyrin-3 (Ank-3) binding and appropriate trafficking^20–22^. The mapped CKII phosphorylation site in β-ENaC also lies within a similar conserved anchor motif^19,23^. Though Na_V_, KCNQ and ENaC channels are unrelated structurally and genetically, the prior two may provide important clues about the mechanism underlying CKII regulation of ENaC. As mentioned above, phosphorylation of Na_V_ and KCNQ by CKII is necessary for Ank-3 to bind to these channels and influences their cellular locale and activity. While the physiological importance of the putative anchor motifs within ENaC remains obscure; recently, Ank-3 has been shown to modulate ENaC activity in a cultured murine principal cell model^23^. Furthermore, the specific CKII inhibitor, TBB (4,5,6,7-tetrabromobenzotriazole)^24,25^, significantly decreases the activity of ENaC and elevates sodium excretion *in vivo*^26^.

Such observations led us to hypothesize that phosphorylation by CKII of a key serine residue in a consensus CKII site contained within the canonical anchor motif within the COOH-terminal domain of β-ENaC is necessary for the channel to bind Ank-3 with the latter being necessary for appropriate channel locale and activity, which is required for the physiological function of the channel and the appropriate fine-tuning of renal Na^+^ excretion. To test this hypothesis, we combined whole animal physiology with electrophysiology and a fluorescence imaging strategy to quantify renal Na^+^ excretion, ENaC activity and channel expression levels in the plasma membrane, respectively. Findings show that inhibition of CKII decreases ENaC activity and plasma membrane levels; whereas overexpression of CKII increases ENaC activity and membrane levels. In isolated kidney tubules, ENaC activity is significantly decreased in principal cell-specific Ank-3 KO (PC-specific Ank-3 KO) mice. This results in these KO mice excreting a Na^+^ load faster. Moreover, disrupting the key Ser residue in the CKII phosphorylation site within the anchor motif in β-ENaC decreases channel trafficking to the cell surface and activity. These results are consistent with CKII phosphorylation of ENaC functioning as a molecular switch that favors Ank-3 binding to the channel facilitating channel expression in the plasma membrane and dependent increases in channel activity.

## Materials and methods

### cDNA constructs and cell culture

African green monkey kidney fibroblast-like cell (COS-7) and Chinese Hamster Ovary (CHO) cells were purchased from ATCC (American Type Culture Collection, ATCC, Manassas, VA, USA). COS-7 cells were cultured in Dulbecco's modified Eagle's medium supplemented with 10% fetal bovine serum and 1% penicillin–streptomycin. CHO cells were cultured in Ham’s F-12 K Nutrient Mixture (Kaighn's Mod.) supplemented with 10% fetal bovine serum and 1% penicillin–streptomycin. All cells were incubated at 37 °C in a humidified incubator supplying 5% CO_2_. For total internal reflection fluorescence-fluorescence recovery after photobleaching (TIRF-FRAP) experiments (see below), COS-7 cells were plated on glass bottom MatTek dishes (35 mm petri dish-14 mm microwell, MatTek Corporation, Massachusetts, USA) and transfected with plasmids encoding α-, β-, and γ-mouse ENaC subunits genetically linked to NH_2_-terminal eYFP^27^ using Lipofectamine 3000 reagent (L3000015, Invitrogen, Carlsbad, CA, USA) as described in the manufacturer’s protocol. In brief, 60% confluent cells in a 35 mm dish were treated with 2 μg of total plasmid cDNA, and then incubated for 4–5 h. The medium was then removed and replaced with fresh medium. Cells were used within 24–48 h after transfection. For patch clamp recording (see below), CHO cells were plated on coverglass chips treated with 0.01% Poly-L-lysine (Sigma, St. Louis, MO, USA). Plated cells were transfected with plasmids encoding α-, β-, and γ-mouse ENaC subunits genetically linked to NH_2_-terminal eCFP^27^ using Polyfect reagent (Qiagen, Valencia, CA, USA) as in the manufacturer’s protocol. In brief, 60% confluent cells in a 35 mm dish were treated with 2 μg of total plasmid cDNA, and then incubated for 4–5 h. The medium was then removed and replaced with fresh medium. Cells were used within 24–48 h after transfection. Site-directed mutagenesis of the pEYFP-C1-ENaC-Beta (S631A) plasmid was accomplished with the QuickChange Lightning Site-Directed Mutagenesis Kit (Agilent Technologies, Santa Clara, CA, USA). Standard desalted primers were designed using the Agilent online primer design tool: βS631A-ENaC (5′-TGGACACCATGGAGGCGGACAGTGAGGTG and 5′-CACCTCACTGTCCGCCTCCATGGTGTCCA). Prior to transformation into TOP10 competent cells, the DpnI-treated amplification reaction was purified using a PCR clean-up kit (Zymo Research, Irvine, CA, USA). Mutagenesis was confirmed using Sanger sequencing (Psomagen, Rockville, MD, USA). The plasmids encoding pZW6 (CK2alpha) and pZW12 (CK2beta) were from Addgene (Addgene_27086 and Addgene_27088, respectively; Watertown, MA, USA). Cells were treated with 4,5,6,7-Tetrabromobenzotriazole (TBB, 200 nM) 30 min prior to experimentation. Cells were maintained in culture in the presence of 10 μM amiloride replenished daily.

### Creation of the principal cell-specific ank-3 knockout mouse

Target deletion of 190, 270, and 480 kDa Ank-3 spliced forms specifically in renal principal cells was achieved by breeding male floxed (loxP sites in the introns preceding exon 22 and following exon 23) Ank-3 mice with female B6.Cg-Tg(Aqp2-cre)1Dek/J mice. Exons 22 and 23 are critical for Ank-3 splicing and just upstream of the regions coding for the spectrin-binding domains in the protein^28^. Consequently, Cre-mediated recombination deletes spliced forms containing Ank-3 repeats that are required for membrane protein interactions. These larger Ank-3 spliced forms are necessary for polarized expression of E-cadherin in renal collecting ducts^29^. The floxed Ank-3 mouse was a kind gift from Dr. V. Bennett (Howard Hughes Medical Institute, Duke Univ., Durham, NC, USA) and has been described previously^29^. The B6.Cg-Tg(Aqp2-cre)1Dek/J mouse was a kind gift from Dr. D. Kohan (University of Utah Health Science Center, Salt Lake City, Utah, USA) and has been described previously^30,31^.The PC-specific Ank-3 KO line was continued by backcrossing floxed Ank-3:Aqp2-cre female to floxed Ank-3 male mice. Homozygous floxed Ank-3, heterozygous Aqp2-cre (PC-specific Ank-3 KO) mice were used for experiments. Shown in Supplemental Figure S1 are the products of a typical genotyping reaction for these lines (see also below). Wild type with or without Aqp2-cre, and Aqp2-cre-negative littermates lacking or harboring floxed Ank-3 were used as controls. No noticeable difference in behavior, body weight, pathology, or any other gross attribute was observed between experimental and littermate controls.

For genotyping reactions, the WT and floxed Ank-3 genes were identified with the forward 5′-TTAATTTGGGGAGGGGGAGTC-3′ and reverse 5’-TTGGGATGCTTTGATTCAGGG-3’ PCR primers, which are expected to produce 366 and 434 bp products for the WT and floxed genes, respectively. The Aqp2-cre transgene was identified with the forward 5′-CTCTGCAGGAACTGGTGCTGG-3′ and reverse 5′-GCGAACATCTTCAGGTTCTGCGG-3′ PCR primers, producing an expected 673-bp product.

### TIRF-FRAP experiments

To determine whether TBB treatment, CKII over-expression or mutation of β-ENaC affected ENaC plasma membrane levels, we selectively illuminated the plasma membrane with TIRF microscopy as described previously^32^. In brief, fluorescence emissions from membrane eYFP-tagged channel subunits were collected in COS-7 cells at room temperature using TIRF microscopy to selectively illuminate the plasma membrane and thus focus on signals from this cellular locale. TIRF generates an evanescent field that declines exponentially with increasing distance from the interface between the cover glass and plasma membrane illuminating only a thin section (∼100 nm) of the cell in contact with the cover glass, including the plasma membrane^33,34^. Fluorescence emissions from fluorophore-tagged ENaC were collected using an inverted TE2000 microscope with through-the-lens (prismless) TIRF imaging (Nikon, Melville, NY, USA). This system was equipped with a vibration isolation system (Technical Manufacturing Corp., Peabody, MA, USA) to minimize drift and noise. Samples were imaged through a plain Apo TIRF 60 × oil immersion, high resolution (1.45 NA) objective. Fluorescence emissions from tagged subunits were collected with the Chroma Technology Corp. (Bellows Falls, VT, USA) 514 nm laser filter set with band-pass emission (Z514BP) by exciting eYFP with an argon ion laser (80 milliwatts) with an acoustic optic tunable filter (Prairie Technologies, Hutto, TX, USA) used to restrict excitation wavelength to 514 nm. In this system, a 514 nm dichroic mirror (Z514rdc) separates the 514/10 nm (Z514/10 ×) and 560/50 nm (HQ560/50 m) excitation and emission filters. Fluorescence images were collected and processed with a 16 bit, cooled charge-coupled device camera (Cascade 512F; Roper Scientific, Sarasota, FL, USA) interfaced to a PC running Metamorph software (Molecular Devices, San Jose, CA, USA). This camera uses a front-illuminated EMCCD with on-chip multiplication gain. Images were collected with a 200 ms exposure time immediately before and after photobleaching and every subsequent minute.

As described previously^32^, Fluorophore-tagged channels in the plasma membrane were photobleached with TIRF illumination using the argon ion laser (514 nm) at full power (100%) for 10 s. Fluorescence emissions from membrane fluorophores were collected under TIRF illumination before and after photobleaching. Laser power, camera gain, and exposure times were constant throughout the course of the experiment except during photobleaching as noted above.

### Whole-cell patch-clamp recording

Whole-cell macroscopic current recordings of mouse ENaC (mENaC) expressed in CHO cells were made under voltage clamp conditions using standard methods^26,27^. In brief, current through ENaC was the inward, amiloride-sensitive Na^+^ current with a bath solution of (in mM) 150 NaCl, 1 CaCl_2_, 2 MgCl_2_, and 10 HEPES (pH 7.4) and a pipette solution of (in mM) 120 CsCl, 5 NaCl, 2 MgCl_2_, 5 EGTA, 10 HEPES, 2 ATP, and 0.1 GTP (pH 7.4). Current recordings were acquired with an Axopatch 200B (Molecular Devices) patch-clamp amplifier interfaced via a Digidata 1550B (Molecular Devices) to a PC running the pClamp 11 suite of software (Molecular Devices). All currents were filtered at 1 kHz. Cells were clamped to a 40 mV holding potential with voltage ramps (500 ms) from 60 mV down to -100 mV used to elicit current. Whole-cell capacitance, on average 8–10 pF, was compensated. Series resistance, on average 3–6 megaohms, were also compensated. Cells expressing mENaC were evaluated by quantifying macroscopic current in the presence and absence of 10 µM amiloride.

### Animal care and use

As described previously^35^, all animal use and welfare adhered to the National Institutes of Health Guide for the Care and Use of Laboratory Animals. Protocols were reviewed and approved by the Institutional Animal care and Use Committee of the University of Texas Health Science Center at San Antonio. Mice were housed and cared for in the Laboratory Animal Resources Facility at the University of Texas Health Science Center at San Antonio, which is fully accredited by the Association for Assessment and Accreditation of Laboratory Animal Care and licensed by the United States Department of Agriculture. The animal studies were performed in compliance with the ARRIVE guidelines (Animal Research: Reporting in Vivo Experiments).

### Split-open tubule preparation and single-channel patch-clamp electrophysiology

Split-open tubules amenable to patch clamp analysis were prepared as previously described^36^. In brief, principal cell-specific Ank-3 KO and littermate control mouse kidneys were sectioned transversely. Segments of the connecting tubule and cortical collecting duct were manually micro dissected with forceps and adhered to a glass chip coated with 0.01% Poly-L-lysine. The chips were transferred to an inverted microscope, where the top layer of the collecting duct was split open with sharpened pipettes. Single-channel patch-clamp electrophysiology in the cell-attached configuration was then performed in the gap-free mode on the luminal plasma membranes of principal cells in these split-opened tubules. The bath solution contained (in mM): 150 NaCl, 5 KCl, 1 CaCl_2_, 2 MgCl_2_, 5 glucose and 10 HEPES (pH 7.4) and the pipette solution (in mM): 140 LiCl, 2 MgCl_2_, and 10 HEPES (pH 7.4). In some experiments, cortical collecting duct were preincubated for 30 min with 200 nM TBB. Channel activity (open probability, P_o_, multiplied by channel number, N; NP_o_) was calculated as previously described^36^.

### Metabolic cage experiments

PC-specific Ank-3 KO and littermate control mice (~ 3 month old; ~ 25 g body weight, with both sexes used in approximately equal proportion for experiments) were housed socially at three mice/cage in metabolic cages (Techniplast, Buguggiate, Italy). Metabolic cage studies followed previously published protocols with minor modifications^37^. In brief, *ad lib* access to water and food was allowed for 3 days. In some instances, after this acclimation period mice were injected (i.p.) with 100 µL of a 0.9% NaCl solution (Na^+^ load). Following this injection, urinary volumes and Na^+^ concentrations were measured every 3 h over a period of 9 h. From this, Na^+^ excretion (UNaV) was calculated and the (cumulative) percent of the Na^+^ load excreted reported. In another set of experiments, mice were switched after acclimation to and subsequently maintained with a nominally sodium-free diet (0.01–0.02% Na^+^; TEKLAD, custom diet TD 90,228, Envigo, Indianapolis, IN, USA). Following a period of 7 days with this sodium-free diet, mice were injected (i.p.) with a single bolus of 125 μg/kg TBB in 100 μL of 10% DMSO/sterile water. Every 24 h over the course of the experiment, urine volume, urinary Na^+^ concentration and consumed water were measured. Urinary Na^+^ concentration was quantified with a flame photometer (Jenway, Staffordshire, UK).

### Statistics

Data were analyzed and plotted using GraphPad Prism 9 (GraphPad Software, Inc., San Diego, CA, USA). All summarized TIRF-FRAP data reported as the mean ± standard error of the mean (SEM), as normalized to pre-bleach emission levels (relative FRAP). Summarized whole cell current data reported as the mean ± SEM of the amiloride sensitive current density (at -100 mV). Summarized gap-free single channel data reported as mean ± SEM of NP_o_. Summarized metabolic cage data reported as mean ± SEM of sodium excretion. Summarized data were compared by the Student's (two-tailed) *t* test or a one-way ANOVA. P ≤ 0.05 was considered significant.

## Results

### CKII is necessary for ENaC trafficking to the plasma membrane and channel activity

**Figure 1:**
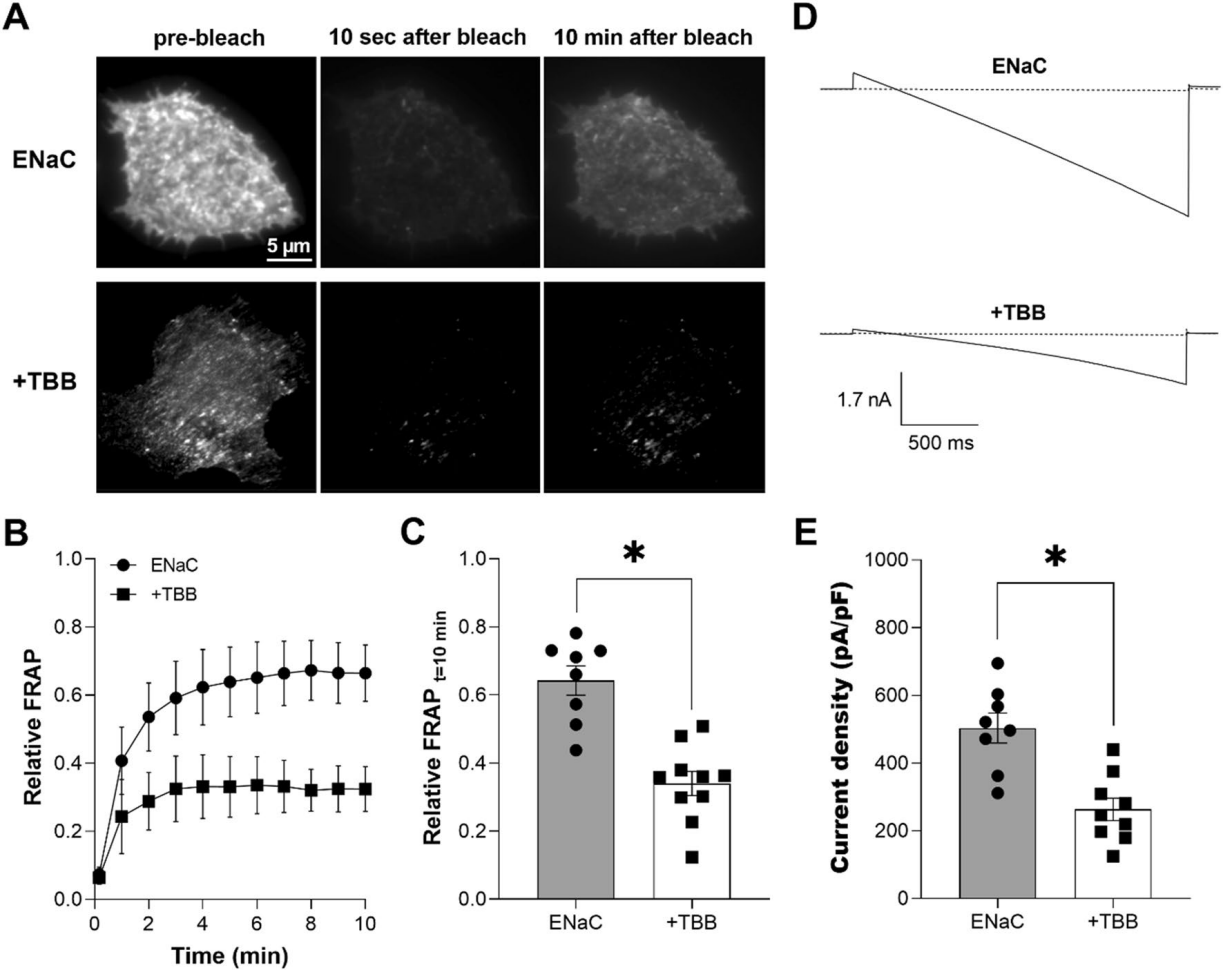
CKII activity is necessary for ENaC trafficking to the plasma membrane and channel activity. (**A**) Fluorescence micrographs of COS-7 cells expressing eYFP-ENaC in the absence (top) and presence (bottom) of TBB treatment prior to photobleaching (left) and 10 s (middle) and 10 min (right) after photobleaching. Cells were treated with 200 nM TBB for 30 min. Images were collected with TIRF microscopy. (**B**) Time course of relative FRAP at the plasma membrane for cells expressing eYFP-ENaC in the absence (black circles) and presence of TBB (black squares). Summary data from experiments (n = 8–10 cells from 2–3 distinct transfections) identical to those shown in (**A**). (**C**) Summary graph of relative FRAP 10 min after photobleaching in cells expressing eYFP-ENaC in the absence (black circles, gray bar) and presence (black squares, white bar) of TBB. Summary data from experiments identical to those shown in (**A**). *P < 0.05 vs. absence of TBB. (**D**) Overlays of typical macroscopic current traces from representative CHO cells expressing mENaC in the absence (top) and presence of TBB (bottom) before and after 10 µM amiloride (dotted lines). Cells were treated with 200 nM TBB for 30 min prior to the recording. Currents elicited by voltage ramps stepped from a holding potential of 40 mV to 60 mV and ramped to − 100 mV. (**E**) Summary graph of ENaC activity (amiloride-sensitive current density at -100 mV) quantified in whole-cell voltage clamped CHO cells transfected with mENaC in the absence (black circles, gray bar) and presence (black squares, white bar) of TBB. Summary data from experiments (n = 8–9 cells from 3–5 distinct transfections) identical to those shown in (**D**). *P < 0.05 vs. absence of TBB.

We began this study by testing whether CKII activity is necessary for ENaC trafficking to the plasma membrane and channel activity. We did this by quantifying ENaC trafficking to the plasma membrane using FRAP combined with TIRF illumination to focus on events at the plasma membrane. To quantify CKII dependent changes in channel activity, we measured macroscopic ENaC activity with the patch clamp technique. Figure 1A shows representative fluorescence micrographs of the plasma membrane before (first panel), 10 s after (middle panel) and 10 min after (last panel) photobleaching of COS-7 cells expressing eYFP-ENaC in the absence (top) and presence of the specific CKII inhibitor, TBB (bottom). Cells were pretreated with 200 nM TBB for 30 min prior to the experiment. Figures 1B, C, respectively, show a diary plot of the time course of relative FRAP and a summary graph of relative FRAP 10 min after photobleaching of ENaC expressed in COS-7 in the absence (black circles, gray bar) and presence (black squares, white bar) of TBB. TBB significantly slowed ENaC trafficking to the plasma membrane with relative FRAP at 10 min being 0.64 ± 0.04 (n = 8) in the absence and 0.33 ± 0.03 (n = 10) in the presence of TBB (*P < 0.05). These results demonstrate that inhibition of CKII slows trafficking of ENaC to the plasma membrane. To test whether this affects channel activity, we quantified macroscopic ENaC currents in CHO cells overexpressing the channel in the absence and presence of TBB. Figure 1D shows typical ENaC currents in the absence (top) and presence (bottom) of pretreatment with TBB before (solid lines) and after 10 µM amiloride (dashed lines). Cells were pretreated, as above, with 200 nM TBB for 30 min prior to the experiment, and amiloride was applied acutely to isolate the amiloride-sensitive macroscopic ENaC current. As shown in the summary graph in Fig. 1E, TBB significantly (*P < 0.05) reduced ENaC activity (reported as current density at -100 mV) from 503.3 ± 44.08 (black circles, gray bar, n = 8) to 263.3 ± 33.12 (black squares, white bar, n = 9) pA/pF. TBB had no effect on capacitance with capacitance being 7–10 pF in the absence and 7–11 pF in the presence of TBB. In these experiments, capacitance was quantified so that whole cell macroscopic currents could be normalized for cell size. A change in capacitance would have indicated a change in cell size or membrane composition, or a change in the integrity of the patched membrane. These results are consistent with CKII activity being necessary for ENaC trafficking to the plasma membrane and dependent channel activity.

### Over-expression of CKII is sufficient to increase ENaC trafficking to the plasma membrane and channel activity

**Figure 2:**
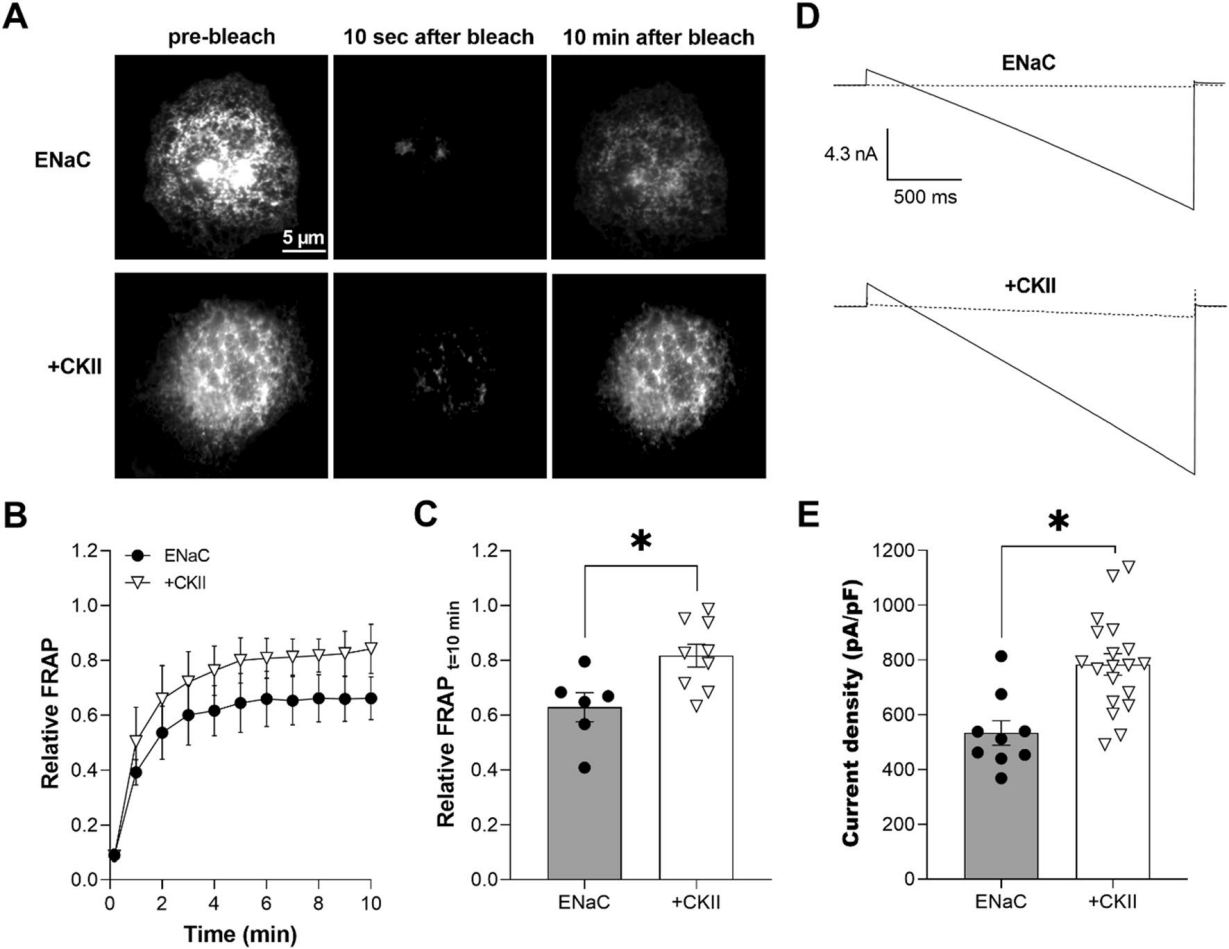
Over-expression of CKII is sufficient to increase ENaC levels in the plasma membrane and activity. (**A**) Fluorescence micrographs of COS-7 cells expressing eYFP-ENaC in the absence (top) and presence of over-expression of CKII (bottom) prior to photobleaching (left) and 10 s (middle) and 10 min (right) after photobleaching. Cells were transfected with eYFP-ENaC and CKII plasmids, CK2alpha and CK2beta. Images were collected with TIRF microscopy. (**B**) Time course of relative FRAP at the plasma membrane for cells expressing eYFP-ENaC in the absence (black circles) and presence of over-expression of CKII (white triangles). Summary data from experiments (n = 6–9 cells from 2–3 distinct transfections) identical to those shown in (**A**). (**C**) Summary graph of relative FRAP 10 min after photobleaching in cells expressing eYFP-ENaC in the absence (black circles, gray bar) and presence (white triangles, white bar) of over-expression of CKII. Summary data from experiments identical to those shown in (**A**). *P < 0.05 vs. the absence of CKII over-expression. (**D**) Overlays of typical macroscopic current traces from representative CHO cells expressing mENaC in the absence (top) and presence of over-expression of CKII (bottom) before and after 10 µM amiloride (dotted lines). Currents elicited by voltage ramps stepped from a holding potential of 40 mV to 60 mV and ramped to − 100 mV. (**E**) Summary graph of ENaC activity (amiloride-sensitive current density at -100 mV) quantified in whole-cell voltage clamped CHO cells transfected with mENaC in the absence (black circles, gray bar) and presence (white triangles, white bar) of over-expression of CKII. Summary data from experiments (n = 9–19 cells from 3–5 distinct transfections) identical to those shown in (**D**). *P < 0.05 vs. the absence of CKII over-expression.

If CKII is necessary for ENaC trafficking and activity, we wondered whether increasing CKII activity would be sufficient to increase channel levels in the membrane, which should also increase channel activity. To test this, we again employed a TIRF-FRAP approach to quantify trafficking combined with electrophysiology to measure ENaC activity in the absence and presence of over-expression of CKII. Figure 2A shows representative TIRF images of COS-7 cells expressing eYFP-ENaC in the absence (top) and presence of over-expression of CKII (bottom) prior to photobleaching (first panel), and 10 s (middle panel) and 10 min after (last panel) photobleaching. Cells were co-transfected with eYFP-ENaC and CKII plasmids, CK2alpha and CK2beta. Figure 2B compares the time course for ENaC recovery in the absence (black circles) and presence (white triangles) of over-expression of CKII. As clear in this figure and summarized in Fig. 2C, over-expression of CKII significantly promoted ENaC trafficking to the plasma membrane with relative FRAP at 10 min being 0.62 ± 0.05 (n = 6) in the absence (black circles, gray bar) and 0.82 ± 0.04 (n = 9) in the presence (white triangles, white bar) of over-expression of CKII (*P < 0.05). We tested next the effects of over-expression of CKII on ENaC activity in whole-cell voltage clamped CHO cells transfected with mENaC. Figures 2D shows typical ENaC current traces in the absence (top) and presence (bottom) of over-expression of CKII before (solid lines) and after 10 µM amiloride (dashed lines). Over-expression of CKII significantly (*P < 0.05) increased ENaC activity (amiloride-sensitive current density at − 100 mV) from 534.1 ± 45.11 (black circles, gray bar, n = 9) to 783.9 ± 39.47 (white triangles, white bar, n = 19) pA/pF (Fig. 2E). Over-expression of CKII had no effect on capacitance with capacitance being 5–8 pF in the absence and 6–10 pF in the presence of over-expression of CKII. These observations are consistent with activation of CKII being sufficient to facilitate ENaC trafficking to the plasma membrane, which in turn increases channel activity.

### Elimination of the CKII phosphorylation site in ENaC impedes channel trafficking to the membrane and decreases channel activity

**Figure 3:**
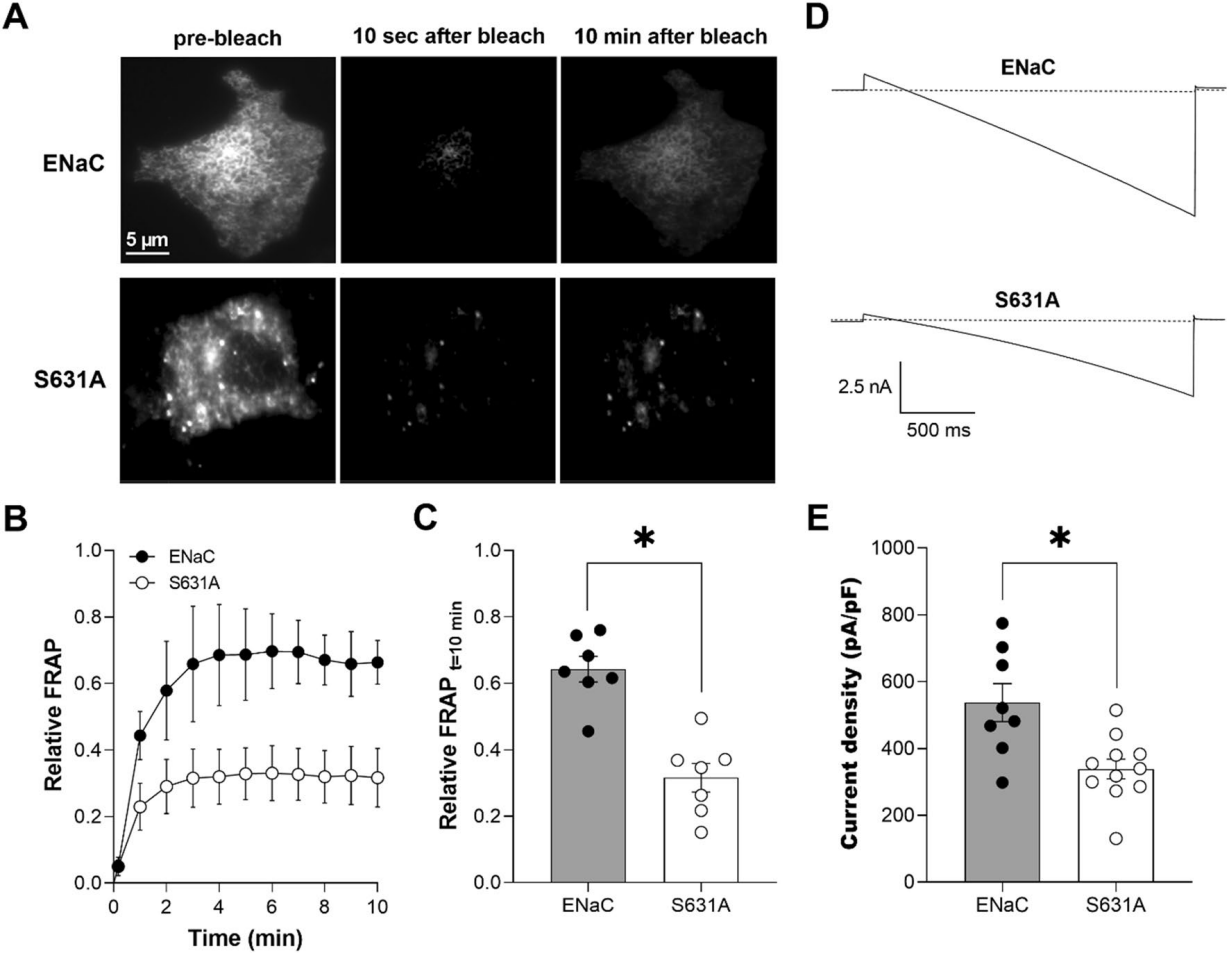
Disrupting the CKII phosphorylation site in ENaC decreases trafficking toward the plasma membrane and channel activity. (**A**) Fluorescence micrographs of COS-7 cells expressing eYFP-ENaC (top) and eYFP-αβ_S631A_γ-ENaC (bottom) prior to photobleaching (left) and 10 s (middle) and 10 min (right) after photobleaching. Images were collected with TIRF microscopy. (**B**) Time course of relative FRAP at the plasma membrane for cells expressing eYFP-ENaC (black circles) and eYFP-αβ_S631A_γ-ENaC (white circles). Summary data from experiments (n = 7 cells from 2–3 distinct transfections) identical to those shown in (**A**). (**C**) Summary graph of relative FRAP 10 min after photobleaching in cells expressing eYFP-ENaC (black circles, gray bar) and eYFP-αβ_S631A_γ-ENaC (white circles, white bar). Summary data from experiments identical to those shown in (**A**). *P < 0.05 vs wild-type ENaC. (**D**) Overlays of typical macroscopic current traces from representative CHO cells expressing mENaC (top) and αβ_S631A_γ-ENaC (bottom) before and after 10 µM amiloride (dotted lines). Currents elicited by stepping voltage from a holding potential of 40 mV to 60 mV and ramping to − 100 mV. (**E**) Summary graph of ENaC activity (amiloride-sensitive current density at -100 mV) quantified in whole-cell voltage clamped CHO cells transfected with mENaC (black circles, gray bar) and αβ_S631A_γ-ENaC (white circles, white bar). Summary data from experiments (n = 8–11 cells from 3–5 distinct transfections) identical to those shown in (**D**). *P < 0.05 vs. wild-type ENaC.

If CKII activity is necessary and sufficient for ENaC trafficking and activity, then disrupting the CKII phosphorylation site embedded within the anchor motif of ENaC should also slow trafficking of the channel to the membrane and decrease channel activity. The site in β-ENaC phosphorylated by CKII has been established with certitude in a prior phosphomapping study^16^, which included mutagenesis of a key Ser residue in the consensus CKII site in β-ENaC to abrogate phosphorylation. To test the necessity of this residue to ENaC activity as regulated by CKII, we substituted S631 in β-ENaC with alanine, which is incapable of being phosphorylated. Figure 3A shows representative fluorescence micrographs of the plasma membranes before (first panel), 10 s after (middle panel) and 10 min after (last panel) photobleaching of COS-7 cells expressing eYFP-ENaC (top) and eYFP-αβ_S631A_γ-ENaC (bottom). Figure 3B shows the time course of recovery of eYFP-ENaC (black circles) and eYFP-αβ_S631A_γ-ENaC (white circles) in the plasma membrane following photobleaching with TIRF illumination. As summarized in Fig. 3C, relative FRAP 10 min after photobleaching in COS-7 cells expressing eYFP-ENaC (0.64 ± 0.04; n = 7) was significantly (*P < 0.05) greater compared to eYFP-αβ_S631A_γ-ENaC (0.32 ± 0.04; n = 7). We observed that the S631A substitution decreased movement toward the plasma membrane in a manner similar to that caused by treatment of wild-type mENaC with TBB (Fig. 1). To complement TIRF-FRAP experiments, we quantified mutant ENaC activity using the whole-cell clamp method. Figure 3D shows typical macroscopic current traces from representative CHO cells expressing wild-type ENaC (top) and αβ_S631A_γ-ENaC (bottom) before (solid lines) and after 10 µM amiloride (dashed lines). The summary graph in Fig. 3E shows a significant (*P < 0.05) reduction in ENaC current (amiloride-sensitive current density at − 100 mV) from 537.3 ± 56.69 (n = 8) to 338.5 ± 29.82 (n = 11) pA/pF in cells transfected with wild-type mENaC (black circles, gray bar) vs. αβ_S631A_γ-ENaC (white circles, gray bar), respectively. Combined these observations are consistent with the conserved CKII phosphorylation site embedded within the anchor motif of β-ENaC modulating ENaC trafficking to the plasma membrane and playing a central role in control of channel activity.

### Deleting Ank-3 specifically in principal cells decreases ENaC activity

**Figure 4:**
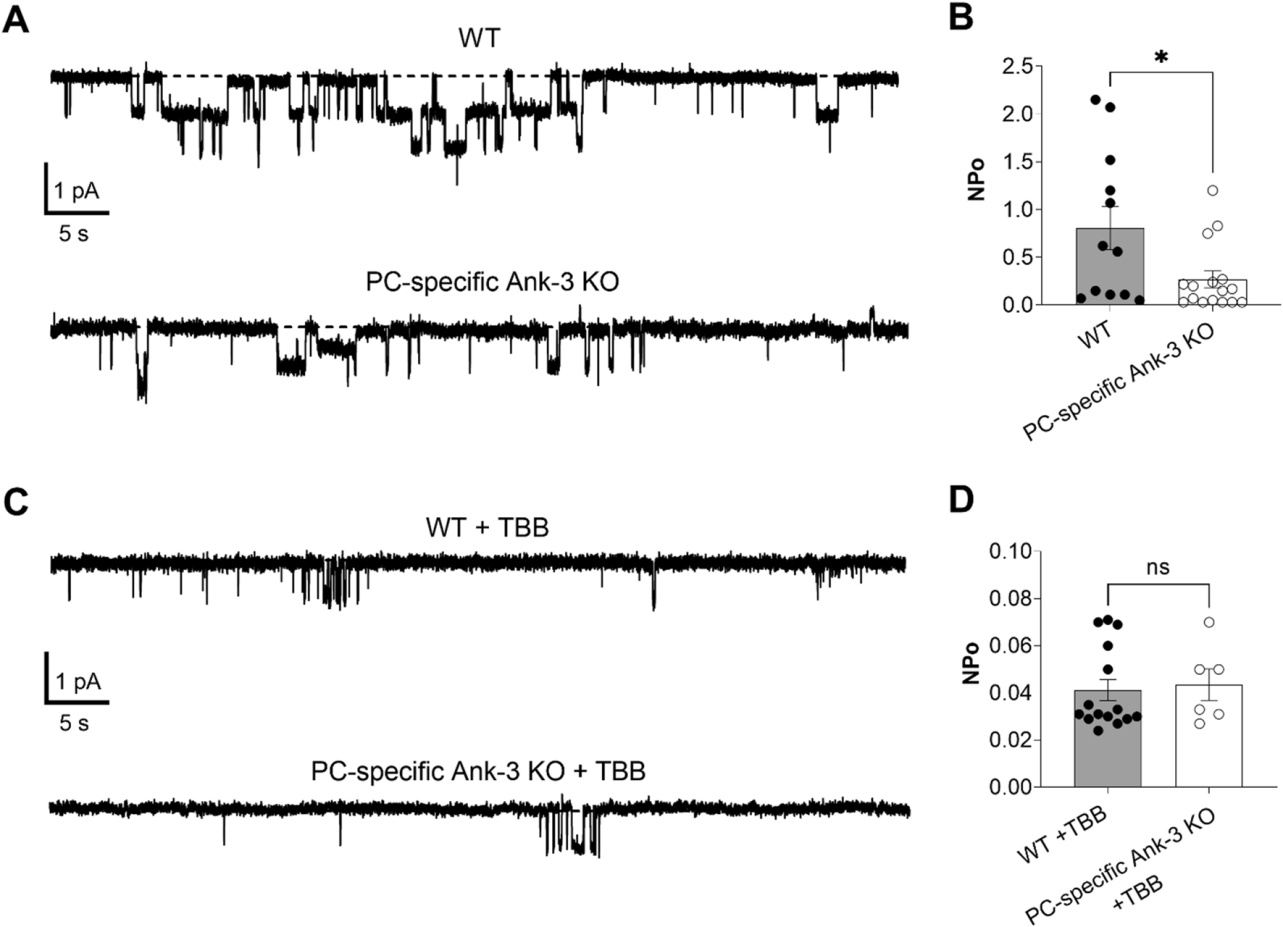
Expression of Ank-3 in principal cells is necessary for ENaC activity in the mouse collecting duct. (**A**) Representative gap-free current traces of ENaC in cell-attached recordings of the apical membranes of principal cells in micro-dissected collecting ducts from WT (top) and PC-specific Ank-3 KO mice (bottom): negative pipette potential -60 mV and inward Na^+^ current is downwards with closed states shown by dashed line. (**B**) Summary graph showing that ENaC activity (NP_o_) is significantly lower in PC-specific Ank-3 KO mice (white circles, white bar) versus WT mice (black circles, gray bar). Data from experiments (n = 12–16 patches from 3–4 distinct mice) identical to that shown in (**A**). *P < 0.05 vs. WT mice. (**c**) Representative current traces of ENaC in cell-attached patches made on the apical plasma membranes of principal cells in isolated tubules from WT (top) and PC-specific Ank-3 KO mice (bottom) pretreated for 30 min with 200 nM TBB. (**D**) Summary graph showing that ENaC activity is not different in tubules from WT (black circles, gray bar) versus PC-specific Ank-3 KO mice (white circles, white bar) pretreated with TBB. Data from experiments (n = 6–15 patches from 2–3 distinct mice) identical to that shown in (**C**).

Because this key CKII phosphorylation site in β-ENaC is embedded within a canonical anchor motif that is conserved across species, and because CKII phosphorylation of similar sites within Na_V_ and KCNQ channels functions as a molecular switch favoring Ank-3 binding and dependent trafficking to proper cellular locales^20,22^, we wondered whether disrupting Ank-3 expression specifically in principal cells would affect ENaC activity and dependent sodium excretion. We tested the necessity of Ank-3 for ENaC activity in native principal cells comparing activity in tubules isolated from WT and PC-specific Ank-3 KO mice. Resting ENaC activity was measured with cell-attached patch clamp electrophysiology in principal cells in freshly isolated connecting tubule and collecting ducts in an unpaired manner comparing activity in PC-specific Ank-3 KO mice with littermate controls. Figure 4A shows typical single channel current traces of ENaC in cell-attached recordings of principal cells in micro-dissected murine tubules isolated from control (top; WT) and PC-specific Ank-3 KO mice (bottom). Negative pipette potential was − 60 mV and inward single channel Na^+^ currents are downwards. ENaC activity, recorded as NP_o_, in the WT mouse was 0.81 ± 0.23 (n = 12). This is significantly (P < 0.05) greater than the 0.27 ± 0.09 (n = 16) measured in PC-specific Ank-3 KO mice (Fig. 4B). The targeted deletion of Ank-3 in the PC-specific Ank-3 KO mice significantly decreased ENaC activity compared to their littermate controls. These findings are consistent with the conclusion that Ank-3 is necessary for ENaC activity in the intact tubule.

Because CKII and Ank-3 are both necessary for ENaC activity, and because they possibly share a common transduction pathway that converges on ENaC, we tested next whether inhibiting CKII with TBB had any additional effect on ENaC activity in PC-specific Ank-3 KO mice. As above, this was tested using patch clamp electrophysiology in native principal cells. Figure 4C shows representative single channel current traces of ENaC in cell-attached recordings of the luminal plasma membranes of principal cells in tubules isolated from control (top; WT) and PC-specific Ank-3 KO (bottom) mice. Tubules were pretreated for 30 min with 200 nM TBB. Negative pipette potential was − 60 mV and inward single channel Na^+^ currents are downwards. As shown in Fig. 4D, the ENaC activity of 0.04 ± 0.002 (n = 15) in WT tubules treated with TBB is not different from the 0.04 ± 0.008 (n = 16) in TBB treated tubules from PC-specific Ank-3 KO mice. However, with this dose and time of treatment, TBB significantly (P < 0.05) decreased the activity of ENaC in tubules from both WT and PC-specific Ank-3 mice as compared to untreated tubules (see 4B).

### Disrupting Ank-3 expression specifically in principal cells increases urinary sodium excretion

**Figure 5:**
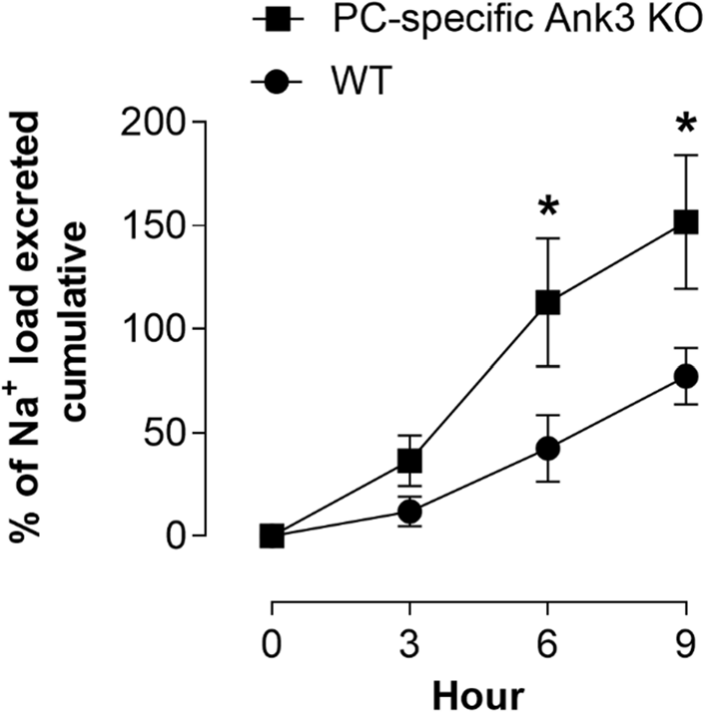
PC-specific Ank-3 KO mice excrete a sodium load faster. Summary graph showing cumulative Na^+^ excretion as a function of time (measured every 3 h) in WT mice (black circles, where n = 4 independent trials) and PC-specific Ank-3 KO mice (black squares, where n = 4 independent trials) injected (i.p.) with a Na^+^ load (100 µL, 0.9% NaCl solution). Load introduced at time 0. Cumulative Na^+^ excretion was significant greater in PC-specific Ank-3 KO mice compared to littermate controls at 6 and 9 h after introducing the Na^+^ load. *P < 0.05 vs. WT mice.

To understand the physiological role played by Ank-3 regulation of ENaC, we quantified the ability of littermate controls and PC-specific Ank-3 KO mice to excrete a Na^+^ load. To do this, mice were injected with 100 µL of a 0.9% NaCl solution. Urinary volumes and Na^+^ concentrations were measured, subsequently, every 3 h over a period of 9 h. Figure 5 shows the cumulative excretion of this Na^+^ load over this period for littermate controls (WT) and PC-specific Ank-3 KO mice. PC-specific Ank-3 KO mice significantly excreted this Na^+^ load faster, where the cumulative percent of the load excreted by 6 and 9 h was greater (P < 0.05) in the PC-specific Ank-3 KO mice compared to WT littermates. These findings are consistent with Ank-3 in principal cells being necessary for the excretion of sodium.

### Ank-3 is downstream of CKII with respect to regulation of ENaC

**Figure 6:**
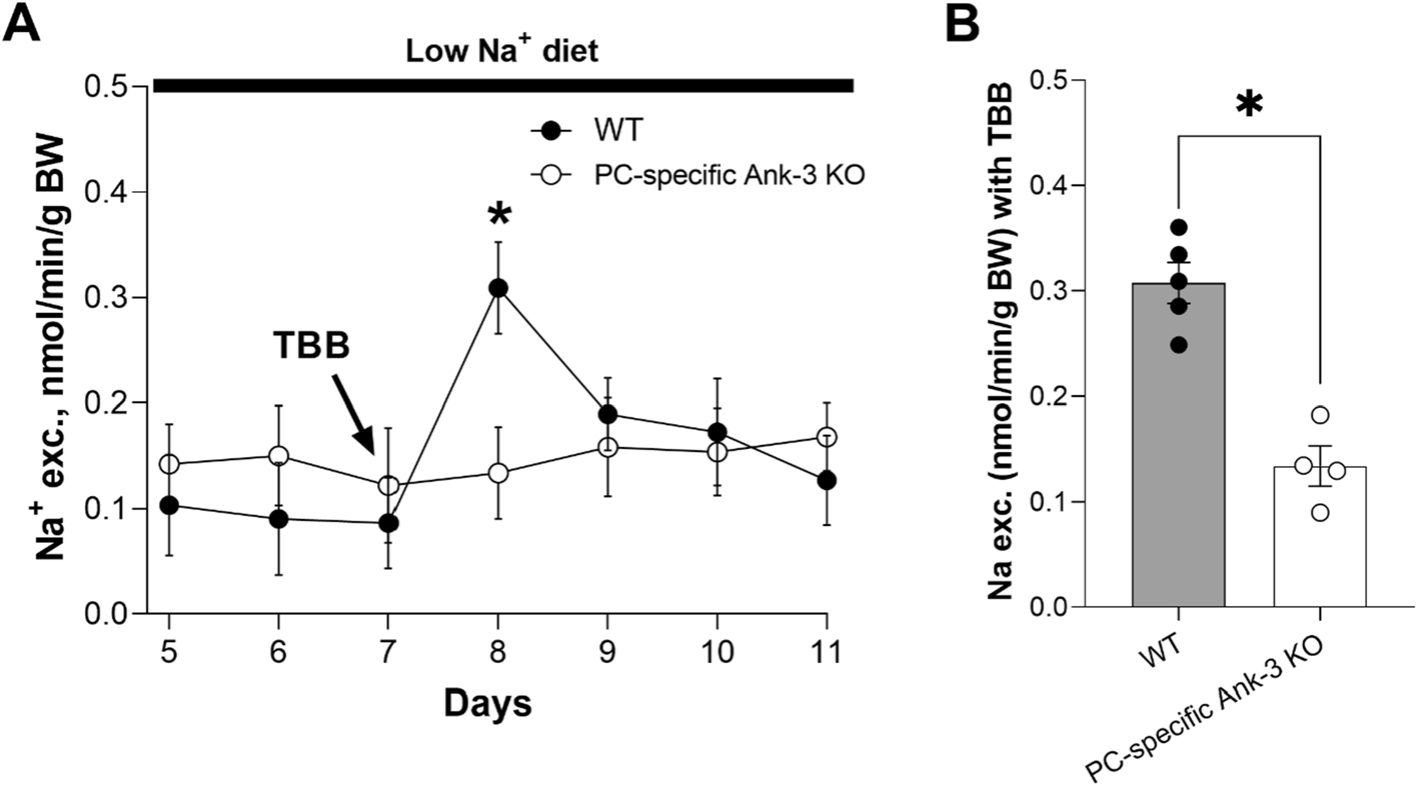
Ank-3 is downstream of CKII with respect to regulation of ENaC. (**A**) Summary graph showing Na^+^ excretion as a function of time in WT mice (white circles, where n = 5 independent trials with three mice per trial) and PC-specific Ank-3 KO mice (black circles, where n = 4 independent trials with three mice per trial) before and after treatment with TBB on day 7 of a sodium free diet. TBB was introduced through a single intraperitoneal injection at 125 µg/kg in 100 µL of 10% DMSO/sterile water. Urinary sodium excretion (U_Na_V) was measured in 24 h intervals. *P < 0.05 vs. KO mice and before treatment. (**B**) Summary graph showing the effects of TBB on Na^+^ excretion (on day 8) in WT (black circles, gray bar) and PC-specific Ank-3 KO mice (white circles, white bar). TBB increased Na^+^ excretion significantly more in WT mice compared to PC-specific Ank-3 KO mice, where it had no effect. *P < 0.05 vs. KO mice.

To test the physiological consequences of inhibiting CKII in WT littermates and PC-specific Ank-3 mice, mice were placed in metabolic cages with free access to water and food for 3 days. After this acclimation period, mice were switched to and subsequently maintained with a nominally sodium-free diet to enhance ENaC activity. Following a period of 7 days with this sodium-free diet, mice were injected (i.p.) one time with 125 μg/kg TBB. Urinary sodium excretion was measured in 24 h intervals. Figure 6A shows Na^+^ excretion as a function of time in the WT and PC-specific Ank-3 KO mouse before and after treatment with TBB on day 7. As shown in the summary graph in Fig. 6B, TBB significantly increased Na^+^ excretion (on day 8) in WT mice (black circles, gray bar) but failed to affect Na^+^ excretion in PC-specific Ank-3 KO mice (white circles, white bar). The WT TBB-treated mouse had a significantly greater increase (P < 0.05) in urinary Na^+^ excretion to 0.31 ± 0.02 nmole/min/g BW (n = 5 independent trials with three mice per trial) compared to 0.13 ± 0.01 nmole/min/g BW (n = 4 independent trials with three mice per trial) for the PC-specific Ank-3 KO treated mouse. These results demonstrate that CKII-sensitive sodium excretion is impaired in PC-specific Ank-3 mice.

## Discussion

The current study investigates the role played by CKII and Ank-3 in the regulation of ENaC. We find, in agreement with prior studies^19,26^, that CKII is necessary and sufficient for ENaC activity. This action of CKII is dependent, at least in part, on a key serine (S631) located within a conserved ankyrin binding motif in the COOH-terminal domain of βxs-ENaC. CKII phosphorylates S631 in β-ENaC^16^. Similar anchor motifs that contain conserved serine targets of CKII are also found in the unrelated Na_V_ and KCNQ channels^20^. In these latter channels, phosphorylation by CKII of key serines within these anchor motifs increases the affinity of Ank-3 binding^20^. Ank-3 binding promotes the proper cellular trafficking and localization of Na_V_ and KCNQ channels^21,22^. As with Na_V_ and KCNQ, CKII is necessary and sufficient for the expression of ENaC within the plasma membrane. Moreover, targeted deletion of Ank-3 in principal cells of the PC-specific Ank-3 KO mouse decreases ENaC activity, increases Na^+^ excretion and abolishes (in some instances) CKII sensitivity. Such results are consistent with phosphorylation by CKII of a conserved Ser embedded within an anchor motif working as a molecular switch to promote Ank-3 binding to ENaC to facilitate channel expression in the plasma membrane in support of channel activity and dependent renal Na^+^ excretion. Because other unrelated channels are regulated in a parallel manner by CKII and Ank-3 through similar key seri nes embedded in analogous anchor motifs, this likely represents an example of convergent evolution.

Figure 1 demonstrates that CKII function is necessary for ENaC activity. Selective inhibition of CKII with TBB decreases the levels of recombinant ENaC expressed in the plasma membrane of CHO cells to decrease channel activity. This is in agreement with our previous work where we demonstrated that TBB increases renal Na^+^ excretion by decreasing the activity of ENaC in principal cells of the murine collecting duct^26^. Furthermore, TBB decreases the activity of recombinant ENaC expressed in *Xenopus* oocytes; and the activity of ENaC in mouse trachea and colon, and in the immortalized cortical collecting duct M1 cell line^19^. Other inhibitors of CKII also inhibit that activity of ENaC expressed in oocytes^19^. Thus, CKII activity is necessary for ENaC activity. It currently is obscure, though, whether such regulation of ENaC by CKII is dynamic, responsive to cell signaling, or trophic, being a house keeping process necessary for channel activity.

Overexpression of CKII with ENaC in CHO cells, as shown in Fig. 2, is sufficient to increase ENaC activity. Overexpression of CKII does this by increasing the expression of ENaC in the plasma membrane. This is in agreement with earlier findings showing that activation of native CKII in *Xenopus* oocytes with polylysine increases ENaC activity^19^. Moreover, overexpression and knockdown of Ank-3 in the immortalized mCCD principal cell line increases and decreases, respectively, the Na^+^ current conducted by native ENaC^23^. Thus, similar to changes in CKII activity, changes in Ank-3 levels are sufficient to change the activity of ENaC, and the expression of this cytoskeleton linker protein is necessary for ENaC activity.

As for Na_V_ and KCNQ channels^20,22^, the link between regulation of ENaC by CKII and Ank-3 is likely to be mediated by a key serine (that is within a canonical CKII phosphorylation site) embedded within a conserved anchor motif localized to the COOH-terminal domain of ENaC subunits. Both the γ- and β-ENaC subunits contain such a serine that is phosphorylated by CKII and conserved across species^16^. The serine in β-ENaC (S631), though, is phosphorylated more strongly by CKII and has a greater effect on ENaC activity when disrupted compared to the similar Ser in β-ENaC^16,19^. Thus, the Ser in β-ENaC appears to be the principle target of CKII in the context of regulation of the channel by this kinase and Ank-3. Indeed, as shown in Fig. 3, mutation of S631 to alanine alone is sufficient to decrease ENaC activity as a result of decreased channel expression in the plasma membrane. While the current results are not definitive in this regard, these consequences of the S631A mutation are likely mediated by disrupting Ank-3 interactions with and regulation of ENaC.

In support of this, are the findings shown in Fig. 4A,B quantifying the activity of ENaC in the apical plasma membranes of principal cells in isolated murine collecting ducts as measured with cell-attached patch clamp analysis of single channel ENaC currents. Targeted deletion of Ank-3 in principal cells results in a decrease in ENaC activity that is similar to that observed here and previously in principal cells from WT collecting ducts treated with TBB^26^. Thus, deletion of Ank-3 parallels inhibition of CKII with respect to regulating ENaC activity.

Whereas deleting Ank-3 in principal cells parallels inhibition of CKII with respect to regulation of ENaC, TBB at the doses and times used in the current studies, as shown in Fig. 4C,D, had a more powerful effect on the channel in the ex vivo patch clamp preparation but not in the excretion studies performed in the intact animal (see Fig. 6; and below). This could indicate possible additional effects of CKII on the channel independent of Ank-3. Alternatively, this may be the result of possible temporal and/or dose-dependent effects of TBB or gene deletion; represent possible partial compensation in the knockout mouse; or be preparation dependent. Recall that only the larger spliced forms of Ank-3 that contain membrane protein interacting domains are deleted in the principal cells of the PC-specific Ank-3 KO mouse^28,29^. Other ankyrin isoforms and the smaller spliced forms of Ank-3, which lack membrane protein binding domains, may still be expressed in the principal cells of the PC-specific Ank-3 KO mouse. Gene deletion, moreover, is a chronic state in the animal that allows time for compensation. In comparison, treatment of isolated tubules with TBB is acute and outside of the animal, and as such, less likely to be compensated. In addition, inhibiting CKII is expected to have broader and more trophic effects on general cellular health as compared to deletion of Ank-3. Regardless of which explanation underpins the observations presented in Fig. 4, deleting Ank3 in principal cells has a profound and significant effect on ENaC activity.

The physiological consequences of inhibiting CKII, and disrupting Ank-3 expression specifically in principal cells is also analogous. As shown in Fig. 5, PC-specific Ank-3 KO mice excrete a Na^+^ load more quickly compared to littermate controls. Mirroring this, inhibition of CKII with TBB forces an inappropriate increase in Na^+^ excretion in mice maintained on a Na^+^-free diet (see Fig. 6 and ref.^26^). Thus, similar perturbations of CKII and Ank-3 result in parallel responses with respect to ENaC at the single channel level, the level of the cell, tissue and whole animal.

In the transduction pathway controlling Na_V_ and KCNQ channel trafficking and activity, Ank-3 is downstream of CKII where phosphorylation of a key Ser by CKII in a common anchor motif enhances Ank-3 binding to these channels to facilitate their trafficking and retention at their proper membrane sites^20–22^. As supported by the results shown in Fig. 6, Ank-3 is also likely to be downstream of CKII with respect to regulation of ENaC. Inhibiting CKII with TBB has no effect on Na^+^ excretion in the PC-specific Ank-3 mouse but increases Na^+^ excretion in littermate controls. This parallels earlier findings where TBB had no effect on ENaC harboring a mutation in the key S631 target of CKII phosphorylation^19,26^.

In contrast to Na_V_ and KCNQ channels, there is little direct evidence, to date, that Ank-3 physically interacts with ENaC. Indirect evidence, though, has been provided by findings that the ankyrin binding protein spectrin associates with ENaC in co-precipitation and pulldown assays^38^, and that Ank-3 expression modulates Na^+^ transport mediated by ENaC^23^. Moreover, the current work and earlier studies of function strongly suggest that Ank-3 likely does directly bind ENaC. We do know, however, that CKII binds to and interacts with ENaC to phosphorylate S631^16,19^. Moreover, we know that Ank-3 expression in principal cells is increased by the steroid hormone, aldosterone^39^. Aldosterone increases the activity of ENaC^15^. It is reasonable to suggest that this increase in Ank-3 expression in response to aldosterone contributes to increases in ENaC activity as mediated by CKII phosphorylation of the channel. If correct, then CKII and Ank-3 mediated regulation of ENaC could be a dynamic response, rather than a trophic response, involved in aldosterone-dependent actions on the channel and Na^+^ excretion.

ENaC trafficking in the current study was quantified with a TIRF-FRAP approach. This approach does not discriminate between trafficking to or from the plasma membrane but rather reports the accumulation of new channels in the plasma membrane. Thus, in the current studies CKII-dependent changes in the levels of ENaC in the plasma membrane could represent effects on trafficking to the membrane or from the membrane, or both. Nonetheless, CKII activity is necessary for ENaC to be in the plasma membrane. This is consistent with previous work by Bachhuber and colleagues^19^ where they showed that TBB decreased the expression of recombinant ENaC in the membrane of *Xenopus* oocytes. In this earlier work, findings were consistent with CKII activity antagonizing the inhibitory effects of the ubiquitin ligase, Nedd4-2, on ENaC. Ubiquitination of ENaC by Nedd4-2 promotes retrieval of the channel from the plasma membrane decreasing channel residency time at this cellular locale^40^. From these findings and findings supporting that CKII was not essential per se for membrane expression of recombinant ENaC in oocytes, these authors concluded that phosphorylation of ENaC by CKII disinhibits inhibition of the channel by Nedd4-2 (facilitated retrieval) to increase channel activity^19^.

**Figure 7:**
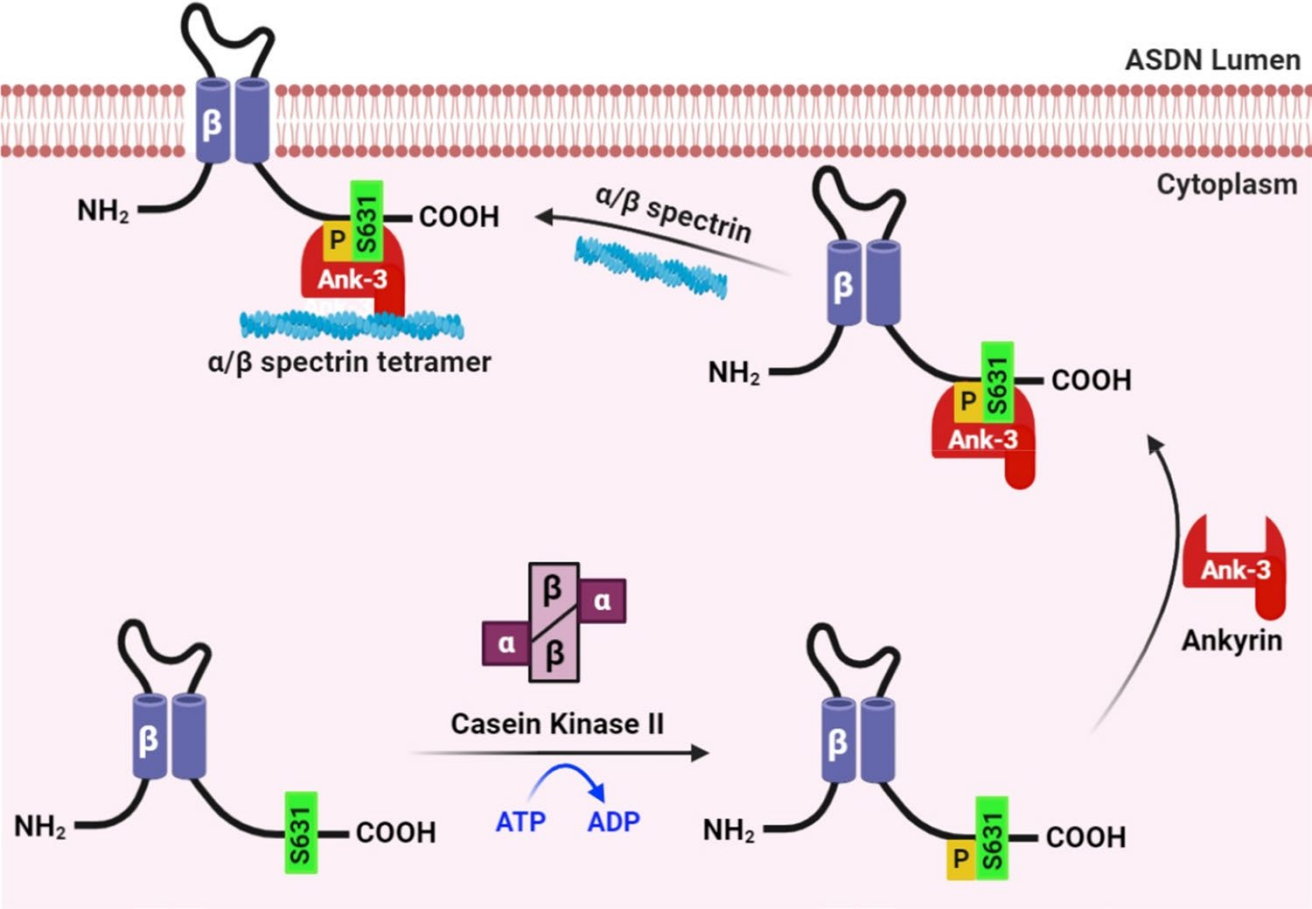
CKII increases ENaC activity by phosphorylating β-ENaC within its anchor motif to promote Ank-3 mediated trafficking to the membrane. Proposed mechanism by which CKII modifies ENaC activity. Our hypothesis is that phosphorylation by CKII of a key serine residue in a consensus CKII site contained within a canonical anchor motif within the COOH-terminal domain of β-ENaC is necessary and sufficient for the channel to bind Ank-3 with the latter being necessary for appropriate channel locale and activity, which is required for the physiological function of the channel and the appropriate fine-tuning of renal Na^+^ excretion. Created with BioRender.com.

The current results and other recent findings regarding regulation of ENaC, Na_V_ and KCNQ channels by CKII-Ank-3 signaling, though, suggest that disinhibition of inhibition by Nedd4-2 is unlikely to be the complete mechanism whereby CKII supports residency of ENaC in the plasma membrane to maintain channel activity. Our current thinking, as summarized in Fig. 7, is that phosphorylation of ENaC by CKII within an anchor motif favors binding of Ank-3 to the channel. It is the interactions between this ENaC bound Ank-3 linker protein with cytoskeleton proteins, such as spectrin, that localizes and stabilizes ENaC within the plasma membrane, in a general sense, without specifically interfering with Nedd4-2 mediated retrieval. This mode of targeting and stabilization is most consistent with how CKII and Ank-3 affects, using a similar CKII-containing anchor motif, Na_V_ and KCNQ channels^20–22^. It is also most consistent with the recent finding that knocking down and overexpressing Ank-3 in immortalized mCCD principal cells decreases and increases, respectively, Na^+^ current mediated by ENaC by increasing the rate of surface delivery rather than slowing the rate of internalization^23^: Nedd4-2 increases the rate of internalization and thus, disinhibition of Nedd4-2 would slow this rate rather than increase the rate of delivery^40^. Nonetheless, CKII and Ank-3 are both necessary for the expression of ENaC in the plasma membrane and channel activity. Neither CKII nor Ank-3, though, affects whole cell expression of ENaC^19,23^. Thus, the effects of the CKII-Ank-3 axis on ENaC then must be through effects on targeting and/or residency of the channel in the plasma membrane.

Effects on ENaC trafficking of this CKII-Ank-3 axis may fit into a larger more general system for sorting plasma membrane proteins in renal collecting duct epithelial cells. Expression of the larger spliced forms of Ank-3 is required for the polar sorting of E-cadherin in collecting duct epithelial cells^29^. Moreover, clathrin-dependent transcytosis recovers E-cadherin mis-sorted to the apical membrane^29^. ENaC, similar to E-cadherin, is affected by clathrin-mediated trafficking and interacts with clathrin-linker proteins^41^. While this emerging evidence suggests that there may be some commonality in the Ank-3 and clathrin-mediated sorting of E-cadherin and ENaC, specific details about these processes remain obscure. These details are likely to explain why ENaC sorts to the apical membrane and E-cadherin to the lateral membrane.

The current findings elaborated understanding of the regulation of ENaC by CKII and Ank-3 increasing our appreciation of the mechanism whereby these proteins control ENaC activity. They also document the physiological consequences of regulation of ENaC by the CKII-Ank-3 axis. Such regulation clearly plays an important role in governing ENaC-dependent Na^+^ excretion. Consequently, regulation of ENaC by the CKII-Ank-3 axis is important to the control of blood pressure.

## Supplementary Information

Supplementary Information.
