# Supplementary material for: Mechanisms and consequences of casein kinase II and ankyrin-3 regulation of the epithelial Na+ channel
Source: Sci Rep. 2021 Jul 16;11:14600. doi: 10.1038/s41598-021-94118-3 (PMC8285517; doi:10.1038/s41598-021-94118-3)
Supplement: Supplementary file 1 — Supplementary Information. [file 41598_2021_94118_MOESM1_ESM.pdf]

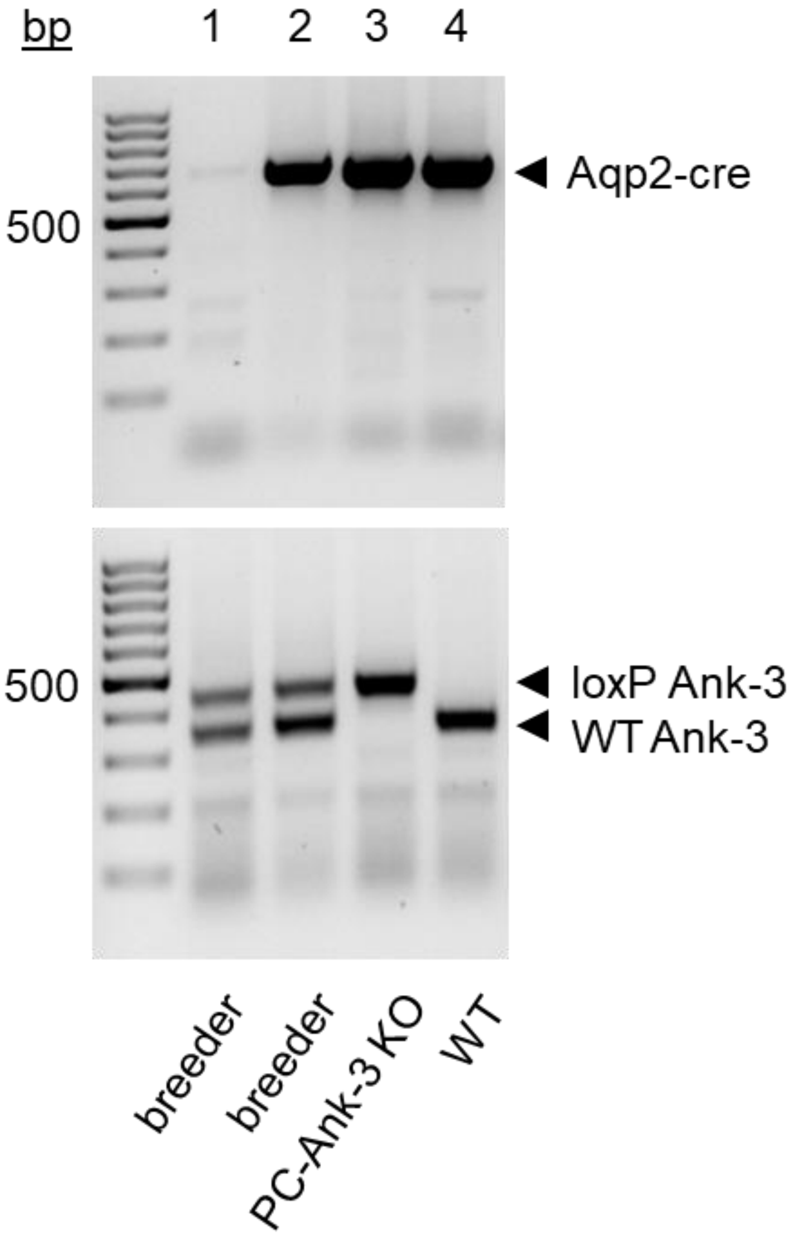

Figure S1. PC-specific Ank-3 KO mice. Micrographs of representative gels containing products from genotyping reactions for breeder (lanes 1 & 2), PC-specific Ank-3 KO (lane 3), and littermate control (lane 4) mice. Products for the Aqp2-cre transgene (top gel), and the floxed Ank-3 and wild type alleles (bottom gel) are indicated with arrowheads. For presentation purposes, contrast and brightness were adjusted, and the image was inverted (black to white) to maximize clarity without changing content. bp = base pairs.
